# The use of digit ratios as markers for perinatal androgen action

**DOI:** 10.1186/1477-7827-4-10

**Published:** 2006-02-26

**Authors:** Matthew H McIntyre

**Affiliations:** 1 Department of Epidemiology, Harvard School of Public Health, 677 Huntington Avenue, Boston MA 02115, USA

## Abstract

Since the ratio of the second-to-fourth finger length was first proposed as a marker for prenatal androgen action in 1998, over 100 studies have been published that have either further tested the association between the digit ratio and prenatal androgens, or employed digit ratios as a marker to investigate the association between prenatal androgens and a variety of outcomes, including behavior, fertility, and disease risks. Despite the clear demand for an adult marker of prenatal androgen action and increased use of digit ratios as such a marker, its validity remains controversial. This review (1) evaluates current evidence for the relationship between digit ratios and prenatal androgens (using experimentation with animal models, amniotic testosterone, and congenital adrenal hyperplasia case-control studies), (2) describes opportunities for future validation tests, and (3) compares the potential advantages and disadvantages of digit ratio measures with more established methods for studying the effects of prenatal androgens.

## Background

Many researchers are pursuing research programmes aimed at elucidating the effects of early testosterone exposure on later development outcomes, especially behavior. These include both experimental work with animal models and a large body of work in human psychology that have focused on the role of early testosterone action on behavioral sex differentiation [1]. In addition to the long-standing work on behavioral outcomes, interest has arisen more recently in the effects of early testosterone exposure on health-related outcomes in adulthood, including polycystic ovary syndrome [2,3] and reproductive cancers [4,5].

The concept of "early testosterone action" is broadly meaningful in mammals because a period of high testosterone production in males during the prenatal (and possibly postnatal) periods drives the differentiation of primary reproductive tissues, and is then followed by a long period of relative testicular quiescence before the onset of puberty. Nevertheless, the particular pattern and timing of early testosterone production varies among species, and, more importantly, the pattern and timing of potential target tissue development varies substantially both between species and among tissue types. Rodents, for example, might be expected to show greater potential effects of postnatal, relative to prenatal, testosterone exposure because of their altricial pattern of development, whereas primate males (including humans) might be expected to show greater postnatal effects because they produce more testosterone in infancy [6]. This review will focus on the pattern and effects of testosterone production in human males, with comparisons to animal models where relevant.

## Human male perinatal testosterone production

The mid-gestational peak in human male testosterone production occurs between the 10^th ^and 18^th ^weeks of gestation. Testosterone levels then decline, probably for a combination of reasons including an increase in hypothalamo-pituitary sensitivity to negative feedback, rising concentrations of placental steroids, and rising prolactin production [7]. Late pregnancy is presumed to be characterized by much lower testicular activity, as reflected by the low testosterone concentration, and sex similarity, found in umbilical cord blood [7-10], though fetal steroid profiles in the weeks immediately preceding birth have not been well characterized. Parturition events cause an LH surge followed after several hours by a surge in testosterone [11]. The proximal causes of the LH surge are not well understood, but may involve the abrupt removal of the feedback effects of placental steroids. Serum total testosterone concentrations in boys fall to childhood levels by one or two weeks after birth, then rise to a second postnatal peak beginning at about 8 weeks, and fall again to childhood levels by 4–6 months [7]. Human perinatal testosterone production, then, consists of three distinct peaks, a mid-gestational peak and two postnatal peaks.

Total serum testosterone levels at each of the peaks rise to just below or within the range of concentrations in adult men. Sex hormone binding globulin concentration is very low at birth, but rises rapidly, such that the unbound level of testosterone is high at the first postnatal peak but low at the second postnatal peak [7]. This is reflected in the high concentrations of testosterone in the saliva of one-week old infants, followed by low concentrations during the infant peak detected in blood [12,13]. Conversely, concentrations of dihydrotestosterone are low in the first weeks after birth but very high during the second postnatal peak [14].

Of the three peaks in human perinatal testosterone production, the mid-gestational peak is known to have important biological effects, more or less directly producing the sex differences observed at birth, but sex differentiation may continue after birth. The testes also grow especially rapidly during infancy, which might reflect testicular activity and gonadotropin stimulation [13,15]. Some preliminary evidence also suggests that postnatal testosterone production in necessary for normal penile growth in infants [16].

## Digit ratios: rationale, methods of measurement, sources of error and confounding

As peripheral blood cannot be drawn from fetuses *in utero*, and is rarely drawn from infants for research purposes, those interested in studying effects of perinatal testosterone in humans have had to rely on a variety of other methods. One method which has come into broad use is finger or digit length ratio measurement, especially the ratio of the second (index)-to-fourth (ring) fingers (2D:4D). 2D:4D was proposed as a marker for *prenatal *testosterone by John Manning and colleagues in 1998 [17].

Given the complexities of early effects of testosterone on sex differentiation discussed above, and further complexities associated with the development of the short bones of the fingers, this review evaluates evidence bearing on the broader hypothesis that digit ratios, including 2D:4D, are influenced by *perinatal *androgen action, and if so, whether the effects are large enough and specific enough to merit their use as biomarkers. The word "prenatal" will be used where effects can be isolated as such, or if not, "perinatal" will be used. Moreover, in some cases it will be possible to refer specifically to "testosterone" or more generally to "androgens." While most research has focused specifically on 2D:4D, as will this review, I will also broaden the discussion to "digit ratios" where appropriate. The review continues with (1) a description of digit ratio measures, including methods of measurement, measurement error, and the development of sex differences, followed by (2) consideration of the potential involvement of perinatal androgens in the development of observed sex differences, then (3) evaluation of each established technique for studying effects of perinatal androgens, its applications to the validation of digit ratio measures, and the potential roles of digit ratio measures to complement the established approach, given their respective advantages and disadvantages for different types of research.

The introduction of digit ratios as possible markers for prenatal androgens [17] follows from the very long-established observation that adult men have longer ring fingers (fourth digits) than adult women relative to the lengths of other fingers [18-22]. Methods of digit measurement have followed standard protocols, with most studies measuring on the skin surface, from photocopies, or from digital scans, and a few using left side hand-wrist radiographs originally obtained to assess bone age. Most researchers have focused digit ratio research specifically on 2D:4D, with a few exceptions, including authors also measuring other sex-dimorphic ratios like middle-to-ring finger, 3D:4D [23-25], and researchers using an older method of assessment, distal extent [22]. In each case, female values are higher. For 2D:4D measured on the skin surface, lengths of each finger are determined from the most proximal flexion crease (as two flexion creases often appear at the base of the fourth digit) to the fingertip. The intra- and inter-observer repeatability of these measures have been established by a number of researchers employing 2D:4D [26].

In adults, the point-biserial correlation between sex and right-hand, skin-surface 2D:4D is not high, even in racially homogenous samples (*r *= 0.22, ~60% overlap between male and female distributions [17]). Furthermore, the large population and racial differences observed in both adults [27-29] and children [25,30] could introduce serious confounding. In mixed race samples, race accounts for more variation in 2D:4D than does sex. African-descended populations have lower 2D:4D at all ages, but substantial differences have also been reported among national or ethnic groups usually considered racially similar [28]. However, the magnitude of sex differences reported in right-hand, skin-surface 2D:4D have been similar in different populations, so sex differences appear to be independent from population differences. The problem of racial confounding can be avoided by careful study design, including collection of homogeneous samples and comparisons within families. Alternative digit ratios, like 3D:4D, might also reduce racial confounding [25].

The size of the sex difference in right-hand 2D:4D seems to be smaller in children than adults (*r *= 0.15, ~90% overlap between boys and girls [17]). However, a longitudinal study using left-hand, radiograph-measured 2D:4D found larger sex differences at 9 years-old (*r *= 0.23) than at 17 (*r *= 0.18) [24]. Sex differences in 2D:4D were shown to be unaffected by puberty using a cross-sectional design [17]. Serial measurements have corroborated the finding [24]. However, it is now well established that 2D:4D increases during childhood in both sexes [24,25,30,31]. The magnitude of the change is small, and in children older than five years old is certainly smaller than the sex difference, but between ages one and five the increase is greater than the sex difference. Age should be considered in planning studies with children.

The development of sex differences in digit ratios during childhood also constitutes an important source of evidence about the role of testosterone in the sex differentiation of digit ratios. Children over the age of one year produce very low levels of sex hormones. Therefore, while sex differences at age five cannot be unambiguously attributed to either mid-gestational or postnatal testosterone production, involvement of testosterone in one or both periods is possible, and involvement of testosterone in later periods is unlikely.

## Possible physiological pathways producing sex differences in digit ratios

Sex steroids could directly influence relative bone lengths either by influencing (1) the development of the phalangeal anlagen during the perinatal period, or (2) metaphyseal growth. In neither case has specific evidence from the short bones of the fingers been reported, and inferred physiology depends on analogy to long bone physiology. Sex steroid receptors are expressed in various fetal anlagen [32]. Influences on the anlagen would occur primarily during the first trimester of gestation, a time of testicular development and rising androgen production levels in the male fetus. Effects of sex steroid exposure on the development of primordia are difficult to speculate except by analogy to the better-understood effects of estrogens on metaphyseal growth. Growth plates organize at one or both ends of long and short bones at various times during fetal or infant development (depending on the particular bone), and account for most elongation. Sex steroids act on metaphyseal tissue primarily through the estrogen receptors α and β [33-36]. In adolescent males effects on long bones are mediated by local aromatization of testosterone [37]. The androgen receptor is also expressed in growth plates [38], though effects on bone length through the androgen receptor are unknown.

The simplest understanding of estrogen-receptor-mediated effects on metaphyseal growth is that they promote fusion of growth plates by the accelerated degeneration of hypertrophic chondrocytes [35]. In addition, sex steroids, again apparently through an estrogen pathway [36], produce the growth spurt associated with the early stages of puberty. The growth spurt seems to result from the systemic role of sex steroids in the increasing secretion of growth factors, especially IGF-1, during puberty [39]. Growth-inhibiting effects of estrogens seem to be dependent on their growth-promoting effects, in that hypertrophic chondrocytes are stimulated by high levels of estrogens, become exhausted, and degenerate as a result [35]. This effect of estrogens on bone growth plays a key role in the normal pattern of linear growth during puberty, including growth in stature. Men attain a higher stature (and generally longer fingers) than women through hypermorphosis, an extended period of growth owing to later onset of the pubertal growth spurt, not through a higher rate of growth. The few men who have been identified with either estrogen receptor deficiency or aromatase deficiency present with continued growth in stature far beyond sexual maturation, and low bone mineral density [40].

Sex steroid effects on anlagen or metaphyseal growth could influence observed differences in relative finger bone lengths in any of a large number of ways, including (1) differences among bones in sex steroid sensitivity, or (2) differences among bones in the sequence of growth processes, overlaid on temporal fluctuations in testicular activity. Sex differences in digit ratios could arise if bones from different fingers are differentially receptive to sex steroids (because they differ in receptor activity, aromatase activity, or present different conditions for interaction between steroid complexes and growth factors). Sex differences could also arise even if the bones of different fingers have similar responses to sex steroids but differ in their temporal patterns of growth. Differences among bones in the timing of periods of peak growth or onset of chondrification or ossification, together with changing sex differences in steroid production, would result in different effects of sex steroids, despite similar patterns of sensitivity and exposure among bones. Differences in the timing of growth events have been extensively documented, both among short bones of the hand [41], and between the sexes [42,43].

These potential explanations are not exhaustive. The effects of perinatal steroids might predispose tissues to divergent patterns of development in later periods. Whether the role of perinatal androgens in promoting sex differences in digit ratios are more direct or indirect could have substantial bearing on the validity of 2D:4D as a specific marker for perinatal androgens.

### Experimental research using animal models

Experimental research into the effects of perinatal androgens in animals has been an important source of insights about effects in humans. Some generalization among mammals with respect to testosterone production and perhaps sensitive periods of some tissues seems warranted. The mid-gestational and first postnatal periods of male testosterone production are common to all mammals thus far studied, but not chickens [44]. The first postnatal peak has been described in rats [45,46], mice [47], horses [44], sheep [48], and in ferrets [49].

However, the value of animal models for studying many outcomes of interest remains limited, including behavioral sex differences [1], metabolic development [50], and metastatic prostate cancer [51], to cite just a few examples. Some problems arise because comparable outcomes are not observed, and other problems arise because differences in physiology or in the timing and patterns of development complicate etiological comparisions. Therefore, methods for studying effects of endogenous perinatal androgens in humans, perhaps including 2D:4D, will remain important.

Could experiments in non-human models be used to test the validity of 2D:4D? Interspecies developmental differences in the digits might also introduce problems with generalization similar to the outcomes mentioned above. Even within primates, *digital formula *can vary substantially. Non-human apes and brachiating monkeys have shorter thumbs and longer fingers, and also lower 2D:4D. But if homologous sex differences could be established in other species, experimentation might help in describing their common origin.

One study found that a similar sex difference in digit ratios is found in weanling and adult laboratory mice [52], which might suggest a taxonomically broad homologous system where sex differences are concerned. A similar sex difference has also been found in the ratios of Hamadryas baboon metacarpals [53], but an opposite sex difference has been reported in 2D:4D of living Guinea baboons [54]. One study has investigated effects on rat digit ratios of prenatal exposure to alcohol, which is known to decrease perinatal testosterone levels in rats [55]. 2D:4D was not significantly altered by the intervention. Research has also proceeded in birds, in which generalization to humans should be more problematic. One study found sex differences in digit ratios in zebra finches [56], but a later study failed to replicate the sex difference [57]. Another study manipulated yolk testosterone in ring-necked pheasant eggs and showed an increase in female 2D:3D on the left foot only [58]. The finding is especially difficult to interpret because 2D:3D is not one of the more sex dimorphic ratios in humans and was not found to be sex dimorphic among the control pheasants. Furthermore, the effect was opposite the expected direction and only on the left foot.

Further research into plausible animal models for digit ratio development, and experiments involving fetal androgen manipulation, might contribute substantially to assessing whether sex differences in 2D:4D result from testosterone action, and if detected, perhaps to describing the magnitude and dose-responsiveness of the effect, particular pathways of action, and confounding factors that could interfere with the use of 2D:4D as a marker. However, further work must first establish whether useful animals models exist, including careful characterization of population mean digit ratios and the development of sex differences.

## Direct measurement of fetal testosterone

As already noted, an important obstacle to the validation of 2D:4D or any purported adult marker for perinatal androgen action, is the lack of peripheral blood sampling from fetuses *in utero*. However, a number of alternative methods for direct measurement of fetal androgen levels have been proposed as useful research tools and one has been applied to validating 2D:4D.

### Amniotic fluid

The mid-gestational surge in male testosterone can be identified in amniotic fluid [10,59-63], though the sex difference is smaller than in fetal peripheral blood, as measured in abortuses, and the correlation between amniotic testosterone level and peripheral blood level is low [64]. Use of amniotic fluid to study prenatal testosterone also has several practical disadvantages associated with the use of amniocentesis in the clinical setting. Samples are always opportunistic, gestational age cannot be carefully controlled, and samples cannot be easily collected to test the validity of amniotic testosterone, the last such test having been conducted 20 years ago with negative results [64].

One study has used amniotic testosterone to test the validity of digit ratios [65]. Non-significant trends were found in a group of 29 boys and girls for amniotic fluid testosterone to be negatively related to right-hand, but not left-hand, 2D:4D at two years old, and for estradiol to be positively related. The ratio of testosterone to estradiol in amniotic fluid was a significant predictor and explained just over 20% of the variance in right-hand 2D:4D. However, the biological significance of estradiol, and of the testosterone-to-estradiol ratio, in prepubertal sex differentiation is not widely supported by other evidence [40,66]. Bone growth, in which estrogen mediation seems to play an important role, might be an exception.

### Neonatal cord blood

A second approach to direct measurement employs neonate umbilical cord blood concentrations of testosterone in the study of perinatal exposure. As already noted, levels of testosterone in cord blood are very low and not correlated with testosterone levels in important periods [7-10]. That said, sex differences in cord testosterone have been detected [67]. Umbilical cord blood has not yet been used to investigate the validity of digit ratios, and would probably not be promising.

### Gravid maternal blood

A third approach to direct measurement uses gravid maternal testosterone levels to study the effects of androgens in fetuses. This approach is even more controversial because the association between maternal and fetal sex steroid levels is not well understood. Gravida hormone levels provide little, if any, information about fetal production [10,68,69], but one study found significantly higher testosterone in women carrying male fetuses [70]. Conversely, indirect evidence suggests that maternal production of androgens may have detectable effects in female fetuses [71,72]. This method has not been used to test the validity of digit ratios, and while positive results would be supportive of digit ratio validity, negative results certainly could not be construed as evidence against the role of androgens in digit ratio sex differences.

Setting aside concerns about the validity of the direct methods of measuring fetal androgens, digit ratios have a number of practical advantages over all direct methods and would serve as an important supplemental measure. While direct measures can be useful for studying outcomes in infancy and childhood, they are less practical for studying outcomes in adulthood, such as cancer or cardiovascular disease, which would require very long, expensive prospective studies. Digit ratios can also be measured and re-measured easily and reliably. While digit ratio studies require larger numbers of subjects to detect effects than do amniotic testosterone studies, digit ratios measures can be collected from many subjects easily, at later ages, and in non-clinical settings, allowing for larger, more controlled, and more representative samples.

## Co-twin sex

Transfer of testosterone from male fetuses to neighboring fetuses via amniotic fluid perfusion has been demonstrated in rodents [73,74], and a similar phenomenon has been proposed as occurring in human twin pregnancies [75]. There is no direct evidence to support this claim. Even if transfer does occur in humans, the effect is small, as notable masculinization of girls with a male co-twin has not be observed. Some studies have found evidence of masculinization of girls with male co-twins [76,77], but not others [78].

A recent study has compared the left- and right-hand 2D:4D of boys and girls, ranging in age from 4 to 15, who were in opposite- or same-sex twin pregnancies [79]. They found that girls with a male co-twin had lower 2D:4D than girls with a female co-twin, but only on the left hand (point-biserial *r *= 0.44, ~45% overlap). Testosterone exposure via amniotic fluid might explain the finding.

## Congenital adrenal hyperplasia

Congenital adrenal hyperplasia (CAH) treated soon after birth has been the gold-standard method for studying effects of prenatal or perinatal androgens. CAH usually results from deficiency in 21-hydroxylase, which converts progestogens into corticoids. Beginning around the 8^th ^week of gestation, excess adrenal progestogens are shunted into the androgen pathway, resulting in elevated concentrations of adrenal androgens: DHEA, DHEA-S, and androstenedione. Corticosteroid treatments are effective at reducing androgen production in females and start immediately upon diagnosis. In the past, genital sex reassignment surgery was almost always performed on genitally inter-sex XX individuals affected with CAH, who were then raised as girls. Extensive research has been conducted into the possible role of prenatal androgen exposure in both the gender identity and sex-typed behavior of CAH-affected people, with conflicting results [80,81].

One study found that 13 females and 7 males with postnatally-treated CAH had lower 2D:4D (but only on the right hands of females and left hands of males) than 44 female and 28 male relatives unaffected by CAH [82]. Another study found that 27 females and 9 males with postnatally-treated CAH had lower 2D:4D (on both hands of females and only the right hands of males) than 52 female and 52 male age-matched controls unaffected by CAH [83]. However, a study employing 2D:4D measured on radiographic films of the left hand failed to find a significant difference between 66 CAH females and 69 age-matched control females and 77 males, though 2D:4D was intermediate between means for unaffected males and females [31]. All three studies used subjects of widely varying ages, but two studies matched cases and controls by age. Given that 2D:4D levels increase with age in both male and female children, it is possible that additional error introduced by using subjects of such varying ages, non-consanguineous controls, or left-hand measures could account for the different results.

Although [31] used the largest number of cases, their design was otherwise less ideal than the two studies finding effects, as they included subjects from ages 1 to 20, encompassing the full period of growth in 2D:4D, apparently matched some cases and controls with age differences greater than one year, and measured only left-hand 2D:4D. [83] included newborns to 13 year olds, also a problematic range of ages, but matched age to within two weeks, and performed analyses separately in subjects younger and older than two years of age. [82] did not use age matching, but all subjects were older than five, by which time 2D:4D is largely established and sex dimorphic, and cases were matched with related controls, further increasing detection power.

While CAH has been the standard model for studying effects of prenatal and infant androgen action, it has some disadvantages not shared by digit ratios. Although researchers try to include only subjects with similar forms of CAH and ages at treatment initiation, it can be difficult to enroll enough cases using strict criteria. Also, as CAH is a disease, cases and controls differ in ways unrelated to androgen exposure, which might be relevant for some outcomes. In cases involving intersex, it is impossible to distinguish effects of androgens from social and personal experiences of the intersex body. Even in cases with surgical modification of the genitalia, some outcomes can be biased by differences in parental behavior toward the child and by clinical interventions themselves, including the experience of the surgery or its effectiveness. As a result, subjects pools (which are small to begin with) can be characterized as more or less unrepresentative of non-clinical populations for many outcomes of interest. A final drawback is that CAH is most useful for studying the effects of perinatal androgens in females because diagnosis and treatment of males is less consistent, whereas, if valid, digit ratios would be useful in both males and females.

## Genetic modulation of sex hormone effects

Genetic polymorphisms that specifically modify androgen action can be used to study effects of androgens on within-sex variation. For example, the amino-terminal domain of the androgen receptor contains a repeated series of CAG (glutamine) elements of variable length. In humans, the repeat is usually 9 to 36 elements long (mode around 20), varying by population. Within the normal range, shorter polyglutamine tracts have been associated, both theoretically and *in vitro*, with enhanced androgen receptor transactivation, and thereby increased masculinization of tissues [84,85].

Higher 2D:4D has been associated with more CAG elements [86], further supporting a direct association of sex differences in digit ratios with androgen action. As 2D:4D was assessed among adults in this study, effects cannot be definitively isolated to the perinatal period, but given the developmental pattern of 2D:4D, pubertal period effects are unlikely. A study relating CAG repeats and 2D:4D among children would serve as more conclusive evidence for perinatal effects of androgens.

While genetic polymorphisms with known functional effects and expression patterns might become important tools for studying the effects of perinatal androgens, as yet only the CAG repeat has shown firm evidence for differential allele function. Moreover, encoded genetic polymorphisms, such as the CAG repeat, affect androgen action at all ages, and would only be applicable to the study of effects of perinatal androgen action for outcomes measured in childhood. As digit ratios develop early and are then fixed, they could be used to study outcomes in childhood and adulthood, using either prospective or retrospective designs (though combining digit ratio measures from children and adults is problematic, as discussed above).

## Conclusion

The validity of digit ratios as markers for perinatal androgen action is supported by a number of lines of recently reported evidence, but further support is needed.

(1) Sex differences in digit ratios develop early in childhood and are unaffected by pubertal growth. This seems to be firmly established.

(2) High amniotic testosterone-to-estradiol ratio predicts low 2D:4D by two years old. While current evidence is supportive, further studies using amniotic testosterone or other direct methods should be conducted.

(3) CAH affected girls have lower 2D:4D than unaffected controls. This observation is not firmly established and further studies could help to resolve inconsistencies in the literature.

(4) The CAG repeat polymorphism of the androgen receptor gene influences 2D:4D as predicted. Further studies could help to confirm this finding.

Questions remain that might also be answered through animal or *in vitro *experimentation, provided that the utility of particular models can be established. For example, which periods of androgen exposure during the perinatal period produce observed sex differences in digit ratios? How do the development of the right and left sides differ to yield such varying results (even among laboratory mice)?

Interesting work has already been done using 2D:4D, and it might one day prove, after further validation, to be a simple, reliable, and broadly accepted method for studying early human sex differentiation in large, representative samples. Furthermore, while interest in early testosterone has been greatest among psychologists, epidemiologists have also begun to investigate the role of early development in disease. Given that more direct measurement of early testosterone levels is unworkable in most epidemiological study designs, further research would be encouraged by the availability of markers such as digit ratios.

## Competing interests

The author(s) declares that he has no competing interests.
